# Transformation of Physical DVHs to Radiobiologically Equivalent Ones in Hypofractionated Radiotherapy Analyzing Dosimetric and Clinical Parameters: A Practical Approach for Routine Clinical Practice in Radiation Oncology

**DOI:** 10.1155/2013/713420

**Published:** 2013-11-19

**Authors:** Zoi Thrapsanioti, Irene Karanasiou, Kalliopi Platoni, Efstathios P. Efstathopoulos, George Matsopoulos, Maria Dilvoi, George Patatoukas, Demetrios Chaldeopoulos, Nikolaos Kelekis, Vassilis Kouloulias

**Affiliations:** ^1^2nd Department of Radiology, Radiotherapy Unit, ATTIKON University Hospital, Rimini 1, Haidari, 12462 Athens, Greece; ^2^Microwave and Fiber Optics Laboratory, Computer and Electrical Engineering, National Technical University of Athens, Iroon Polytechniou 9, Zografoy, 15780 Athens, Greece

## Abstract

*Purpose*. The purpose of this study was to transform DVHs from physical to radiobiological ones as well as to evaluate their reliability by correlations of dosimetric and clinical parameters for 50 patients with prostate cancer and 50 patients with breast cancer, who were submitted to Hypofractionated Radiotherapy. *Methods and Materials*. To achieve this transformation, we used both the linear-quadratic model (LQ model) and the Niemierko model. The outcome of radiobiological DVHs was correlated with acute toxicity score according to EORTC/RTOG criteria. *Results*. Concerning the prostate radiotherapy, there was a significant correlation between RTOG acute rectal toxicity and *D*_50_ (*P* < 0.001) and *V*_60_ (*P* = 0.001) dosimetric parameters, calculated for *α*/*β* = 10 Gy. Moreover, concerning the breast radiotherapy there was a significant correlation between RTOG skin toxicity and *V*_≥60_ dosimetric parameter, calculated for both *α*/*β* = 2.3 Gy (*P* < 0.001) and *α*/*β* = 10 Gy (*P* < 0.001). The new tool seems reliable and user-friendly. *Conclusions*. Our proposed model seems user-friendly. Its reliability in terms of agreement with the presented acute radiation induced toxicity was satisfactory. However, more patients are needed to extract safe conclusions.

## 1. Introduction

Radiotherapy is one of the most commonly used and effective methods for the treatment of cancer. The dose-volume histogram (DVH) has been accepted as a tool for treatment-plan evaluation [1]. In order to have a complete treatment plan, the information about the dose distribution and the anatomic location and its extent should be supplemented by a DVH [2]. The DVH is used ubiquitously and plots delivered dose on the *x*-axis and percent volume of the structure of interest on the *y*-axis. The general shape and area under the DVH curve is essential in determining adequate coverage and homogeneity of dose in the target volume as well as in determining acceptable dose to critical structures. Indeed, the DVH has occupied a central role in modern treatment planning [3]. 

 The “target volume" referred to in DVH analysis can be a target of radiation treatment or an organ at risk close to the target [4]. The DVH is, therefore, an adequate tool for evaluating a given treatment plan or comparing different treatment plans. Moreover, DVHs are useful for evaluating the uniformity of the irradiation on the target volume and on the normal tissues [5]. 

Our study is based on the use of the cumulative DVH, the plot of the volume percentage which receives a specified dose as a function of the dose. It has been proved that the cumulative DVH is more useful and preferred than the differential one [2]. A DVH is computed from physical dose and does not include radiobiological factors. The same DVH will, therefore, be computed for treatment plans whatever size of fraction is used [6]. 

Several studies have been published concerning the hypofractionated irradiation of breast [7–9] and prostate cancer [10, 11], indicating the efficacy of these schedules in terms of treatment outcome and toxicity. Our department has already reported on the clinical outcome of hypofractionated irradiation schedules for either breast or prostate cancer [12–15]. The purpose of this paper was to provide a radiobiological transformation of the conventional DVH, initially for hypofractionated radiotherapy, which should be easy to implement computationally and could be used in the assessment of treatment plans, in the comparison of treatment schedules and in the analysis of radiation side-effects [6].

## 2. Materials and Methods

### 2.1. Radiotherapy Treatment

#### 2.1.1. Breast Irradiation

Inclusion criteria in this study were stage I-II invasive carcinoma of the breast after conservative surgery and axillary lymph node dissection. If adjuvant chemotherapy was indicated, it had to be completed before the start of radiotherapy. 

The exclusion criteria were mastectomy, presence of Paget's disease, presence of autoimmune conditions, previous diagnosis of cancer of the thorax, previous diagnosis of breast cancer and operation with bad overall cosmetic outcome, diagnosis of previous or concomitant malignancies or skin disease, breast size in craniocaudal dimension more than 20 cm (or alternatively less than 2,500 mL), and presence of psychiatric or addictive disorders.

All patients were monitored for acute skin toxicity according to the EORTC/RTOG criteria, during radiotherapy schedule once per week and one month thereafter [16]. The maximum monitored value was taken as the final grading score. The primary outcome measure was radiation induced acute skin toxicity. 

Patients underwent standard CT simulation in the supine position, using an angled breast board. The ipsilateral breast and CT-visible excision cavity (tumor bed) were contoured for the delineation of target volumes, while contralateral breast, left and right lung, and heart were contoured as organs at risk (OARs). The excision cavity was contoured on the planning CT scan and represented the clinical target volume (CTV) with surgical clips defining the extension of the tumor bed. When surgical clips were not present, preoperative mammography and ultrasound data were used for tumor bed definition. The planning target volume of the tumor bed (PTVt) was a 1-2 cm expansion around the excision cavity CTV. The ipsilateral breast volume was the planning target volume (PTVB), excluding the chest wall and 0.5 cm from the skin [17]. The total prescribed physical dose was delivered with 2.66 Gy daily fractions (Monday to Friday) to the whole breast and the tumor bed, given in with 16 and 3-4 fractions, respectively [13, 14].

#### 2.1.2. Prostate Irradiation

Each patient underwent a virtual CT-simulation, in supine position, using “knee sponge” to consistently align thighs. Patients were instructed to have a full bladder and empty rectum (following a dietary suggestion) during simulation and the whole course of treatment. For treatment planning, a CT scan covering a region from the first lumbar vertebra to the lower part of the perineum was obtained for each patient. A conventional virtual CT simulation was performed to define preliminary isocenter and beam width. All contouring of target volumes and normal structures (organs at risk, OARs) were performed in the Prosoma Contouring System (Pi Medical Ltd., Athens, Greece). The final planning with the dose calculations was performed with the Varian Eclipse Treatment Planning System (Palo Alto, CA, USA). 

Magnetic resonance and computed tomography images were obtained at 3 mm intervals. The CT and MRI were registered by the Prosoma system, while corrections were made in the CT-based contouring of the prostate by taking into account the MRI images. CT and MRI images were obtained nearly 4 weeks after balloon implementation in order to avoid the postimplantation oedema.

The following structures were delineated: CTV and PTV according to the ICRU criteria [18–20]. The CTV was the prostatic gland, while the PTV was obtained by expanding CTV with a margin of 1 cm in each direction and of 0.7 cm posteriorly [21]. The CTV, PTV, and OARs were outlined on all CT slices. No patients received pelvic node or seminal vesicles irradiation. Beams were conformally shaped around the PTV and multileaf collimator (MLC) was employed to improve dose homogeneity. To evaluate the dose constraints for normal tissues, we used the NCCN 2010 guidelines, the Radiation Oncology Group (RTOG) GU consensus, as reported by Lawton et al. [22], and the QUANTEC report corrected for hypofractionation [23]. 

The dose constraints for the OARs are described below:

 bladder: *V*_75_ < 25%, *V*_70_ < 35%, *V*_65_ < 50%,
 rectum: *V*_75_ < 15%, *V*_70_ < 20%, *V*_65_ < 25%, *V*_60_ < 35%, *V*_50_ < 50%,
 femoral heads: *V*_50_ < 5%,
 small bowel: *V*_52_ = 0%,
 penile bulb: mean dose < 50 Gy,

where the parameters *V*_*i*_ refer to the percentage of the organ which receives dose *i*, for example, *V*_75_ for the bladder is the percentage of the bladder which receives 75 Gy. 

The PTV was treated, using a four-field technique [24, 25]. The total prescribed physical dose was delivered with 2.75 Gy daily fractions (Monday to Friday) to the whole prostatic gland, given in 21 fractions [15]. Treatments were delivered with 15 MV photon beam generated by a Clinac 2100 C Varian accelerator. 

### 2.2. Software for the Radiobiological Transformation

#### 2.2.1. Software and Data Input/Output

**Figure 1:**
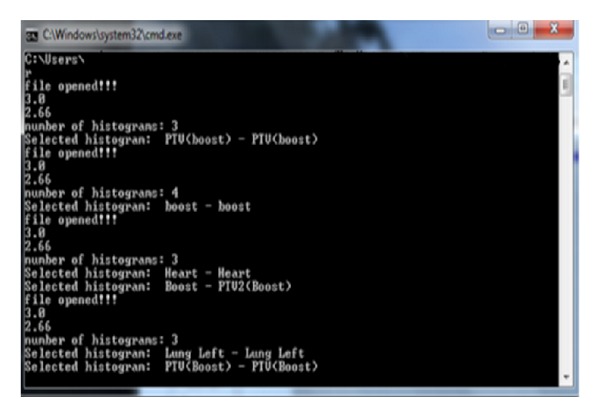
Black box of the software.

The method used in this study was based on the use of a Java software. The cumulative DVH data were exported from Eclipse TPS of Varian Medical System and the DVH data were imported in the Java software for their transformation to their corresponding radiobiologically equivalent DVHs. The black box of the program is shown in Figure 1.

### 2.3. Radiobiological Background

#### 2.3.1. Linear-Quadratic Model

The transformation of physical DVHs to radiobiologically equivalent ones is in agreement with the linear-quadratic model (LQ). The resultant radiobiologically equivalent DVH depends on the value of *α*/*β* ratio, whereas its main difference from the physical DVH is the fractionation size, that is, used in different treatment plans [6]. 

The program received an ASCII file as input and gave the radiobiologically equivalent DVH as output. The ASCII file provided the volume and the OARs percentage, which received a specified physical dose (physical DVH). The transformation is given from the Withers formula, as seen below [26]:

(1) $$\begin{array}{c} {\text{EQD}}_{2}=D\frac{d+\alpha /\beta }{2+\alpha /\beta }, \end{array}$$

where EQD_2_ is the, according to the LQ model, radiobiologically equivalent dose in Gy, *D* is the total dose in Gy, *d* is the dose per session in Gy, and 2 Gy is the conventional dose per session. More specifically, the EQD_2_ is the dose in 2 Gy fractions, that is, biologically equivalent to a total dose *D* given with a fraction size of *d* Gy. The *α*/*β* ratio is different for each tissue. Low *α*/*β* ratios (1–4 Gy) correspond to late-responding tissues, whereas high *α*/*β* ratios (8–15 Gy) correspond to acute-responding ratios [26]. 

**Figure 2:**
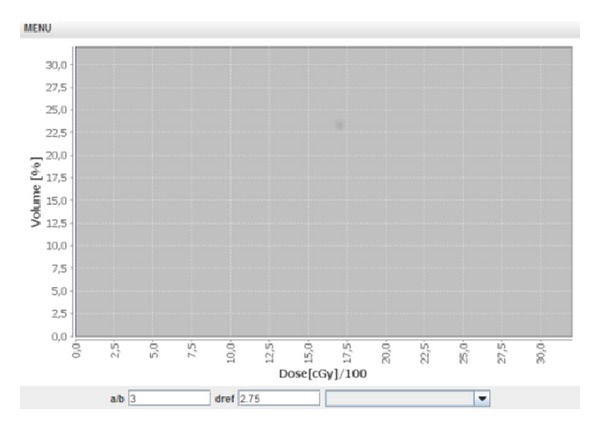
Main menu of the software, where a/b is the *α*/*β* ratio and dref is the dose per session (*d*).

In the present study, the *α*/*β* ratio was defined by the user of the program. More specifically, the *α*/*β* ratio was set to 2.3 Gy for breast edema [27]. The repair capacity of erythema and desquamation is similar, with reported values of *α*/*β* ratio between 7.5 and 11.2 Gy [28]. Thus, we choose *α*/*β* equal to 10 Gy for early responding tissues [26]. In a similar way, we choose *α*/*β* ratio equal to 10 Gy for the rectum, concerning the acute radiation induced rectal toxicity [26]. The selected *α*/*β* ratios for breast and prostate were 4 Gy [29] and 1.5 Gy [30], respectively. Moreover, *d* was set to 2.75 Gy per session for the prostate and 2.66 Gy per session for the breast, while the prescription dose was 57.75 Gy in 21 fractions for the prostate and 53.2 Gy in 20 fractions for the breast. After the values of *α*/*β* ratio and *d* were imported in the program (Figure 2), the radiobiologically equivalent DVH for each patient was taken and analyzed. 

#### 2.3.2. Niemierko Model

In this model, the data taken from the above mentioned ASCII file were converted into the biological equivalent DVH using the equation below:

(2) $$\begin{array}{c} \text{EQDVH}=\int_{i}^{\infty }[(D_{i}\frac{d_{i}+\alpha /\beta }{2+\alpha /\beta }),V_{i}]. \end{array}$$

When *d*_*i*_ < 2 Gy per fraction, then EQDVH is equal to the physical dose; if *d*_*i*_ ≥ 2 Gy per fraction, then EQDVH is referred to the above equation. *D*_*i*_ is the total physical dose in Gy, whereas *d*_*i*_ is the dose per session in Gy. Finally, *V*_*i*_ is the total volume of the organ of interest. The last limitation is necessary so that the Niemierko model is compatible with the LQ model, which is valid only for doses ≥2 Gy and lesser than 7 Gy [31]. 

#### 2.3.3. Radiobiologically Equivalent DVHs

To assess the difference between physical and radiobiologically equivalent DVHs for prostate and breast cancer, we used the data exported from the Eclipse TPS (cumulative physical DVH) and we normalized them using (1) and (2). This process was followed for a sample of 100 patients, 50 patients with breast cancer and 50 patients with prostate cancer. 

### 2.4. Statistical Analysis

For the evaluation of correlations between dosimetric parameters derived from radiobiological DVHs and relevant normal tissue toxicity, we used the Spearman rho nonparametric test. The significant level was set at 0.05. All the analysis was performed by using the SPSS v.10 (IL, USA). 

## 3. Results

**Figure 3:**
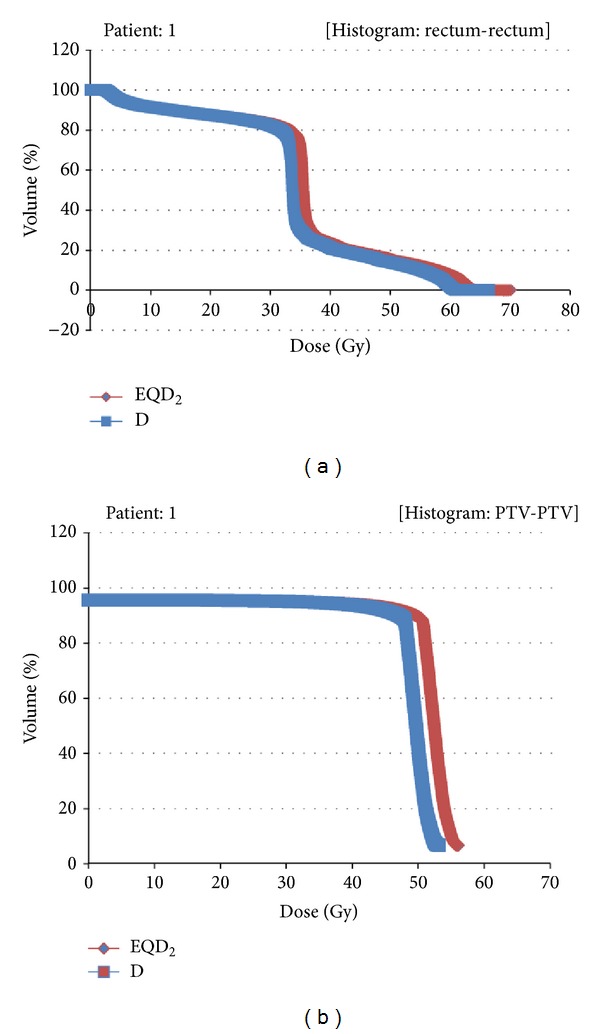
Representative radiobiological equivalent DVHs for the rectum (a) and for the breast (b). The blue plot refers to the physical DVH and the red plot to the radiobiological equivalent one.

The procedure mentioned in Section 2 was applied to all the 100 patients of this study, by means of 100 radiobiological equivalent DVHs derived from the transformation. Two representative radiobiological equivalent DVHs are shown in Figure 3. The average radiobiologically equivalent doses (1) for the rectum and the breast for the different *α*/*β* ratios are shown in Table 1. The patients' characteristics are shown in Table 2 together with the incidence of EORTC/RTOG toxicity for rectal (prostate cases) and skin (breast cases) radiation induced toxicity. 

For the 100 resulted radiobiological equivalent DVHs, dosimetric parameters were evaluated, such as *D*_50_ (Gy) for the rectum, the dose in Gy which receives the 50% of the rectum, *V*_60_ (%) for the rectum, and *V*_≥60_ (%) for the breast. 

In terms of the prostate irradiation, *D*_50_ (Gy) for the rectum for *α*/*β* = 10 Gy was lower than 46 Gy for all the 50 patients. Regarding *V*_60_ (%) for the rectum, when *α*/*β* ratio was equal to 10 Gy, it was lower than 33%. 

In terms of the breast irradiation, *V*_≥60_ (%) for *α*/*β* = 2.3 Gy was lower than 40% for 76% of all the patients, whereas for *α*/*β* = 10 Gy *V*_≥60_ (%) was lower than 40% for 94% of the patients.

Additionally, two statistical analyses were performed, one for the breast and one for the rectum, for the evaluation of correlations between the above mentioned dosimetric parameters derived from the produced radiobiological DVHs and the relevant normal tissue toxicity. The results of Spearman's rho nonparametric test for the rectum, with the significant level, are shown in Table 3. The corresponding results for the breast from Spearman's rho nonparametric test are shown in Table 4.

## 4. Discussion

The present study has shown that the transformation of physical DVHs to radiobiological equivalent ones is a very useful tool, so that the EQD_2_ and the corresponding dosimetric and clinical parameters, for patients that are submitted in Hypofractionated Radiotherapy, are measured. The main aim of this procedure was to estimate the acute toxicity of the adjacent normal tissues. The software constructed for this purpose is user-friendly, especially for the clinicians and can be used in every radiotherapy department since it is a Windows-based software.

Our analysis has shown that the dosimetric parameters, such as *D*_50_ (Gy) and *V*_60_ (%) for the rectum and *V*_≥60_ (%) for the breast, were in some cases higher than the international prescribed dose constraints. According to QUANTEC [28], *D*_50_ (Gy) for the rectum has to be lower than 50 Gy and *V*_60_ (%) has to be lower than 35%. In our study, *D*_50_ (Gy) for *α*/*β* ratio equal to 10 Gy was lower than 50 Gy for all patients. Similarly, the *V*_60_ (%) for *α*/*β* = 10 Gy was lower than 35% for all our patients with prostate cancer (Table 5).

For the patients with breast cancer the *V*_≥60_ (%), according to RSNA [33], has to be lower or equal to 40%. For *α*/*β* = 2.3 Gy, the *V*_≥60_ (%) was lower than 40% for the 76% of all patients. Similarly, for *α*/*β* = 10 Gy the *V*_≥60_ (%) was for the 94% of all patients (Table 5) in accordance with the dose constraints by RSNA [33]. 

The anatomical particularities of some patients, such as breast size, in terms of breast radiotherapy, or rectal volume, in terms of prostate radiotherapy, may have caused the above mentioned deviations in the dosimetric parameters from the international dose constraints. Because of this fact, the irradiation of the adjacent normal tissues, during the treatment planning, could not be avoided and the dosimetric parameters were higher than the prescribed constraints [32, 33]. 

In general, regarding the breast irradiation, previous trials [8, 42] showed no significant differences in efficacy or toxicity between the conventional regimen of 50 Gy in 25 fractions and other hypofractionated regimens, for example, a regimen of 42.5 Gy in 16 fractions. Regarding the prostate irradiation, previous studies [43] have shown that hypofractionation is feasible but is associated with higher rates of acute toxicity compared with patients treated with conventional fractionation.

Regarding the skin toxicity, in terms of breast irradiation, previous studies [44] have shown that a volume receiving >53.9 Gy within PTV (PTV-*V*_107_%) and >55.4 Gy within treated volume (TV-*V*_110_%) were significant predictors of radiotherapy induced skin toxicity. Other studies [40, 45, 46] have analyzed dose-volume parameters, in order to find any possible correlation with acute rectal toxicity during and after conformal radiotherapy for prostate cancer. Valdagni et al. [46] observed that the risk of acute Grade ≥2 Gastrointestinal (GI) toxicity increased significantly with increasing *D*_50_ rectum. During the 3D-RT technique, an increase at the total dose led to an increase of rectal dose *D*_50_. Another study by Matzinger et al. [47] showed that acute rectal toxicity was related to the percent volume of rectum receiving more than 60 Gy (*V*_60_), while Onal et al. [48] observed that acute rectal toxicity was closely associated with *V*_60_ Gy. 

In addition to other similar studies (Table 6), our study also showed that there was a significant correlation between dose-volume parameters, such as *D*_50_ and *V*_60_ and the onset of acute rectal toxicity (*P* < 0.05) for *α*/*β* = 10 Gy (Table 3), for patients who were irradiated with a mean dose of 70 Gy. The interpretation of this correlation is that an increase at the dosimetric parameters *D*_50_ and *V*_60_ leads to an increase at the acute rectal skin toxicity [46–48]. Moreover, the present statistical analysis showed no significant correlation between physical dose and EQD_2_ and the onset of acute rectal toxicity (*P* = 0.881).

Regarding the skin toxicity, the present statistical analysis showed that *V*_≥60_ can be a predictive factor of radiotherapy induced skin toxicity because a significant correlation between the dosimetric parameter *V*_≥60_ and acute skin toxicity (Table 4) was measured (*P* < 0.05). This correlation reveals that an increase at the dosimetric parameter *V*_≥60_ leads to an increase at the grading of acute skin toxicity. 

## 5. Conclusions

Our results suggest that the transformation of physical DVHs to radiobiologically equivalent ones constitutes a useful tool for the clinicians, in terms of important dosimetric parameters, such as *D*_50_ and *V*_60_ for the rectum and *V*_≥60_ for the skin. The tool is also effective and reliable, as far as the significant correlation of dosimetric values with radiation induced acute toxicity is concerned. At last but not least, the main outcome of the statistical significance of spearman rho correlations is that radiobiology was able to predict the relevant acute radiation toxicity. However, more patients are needed to extract safe conclusions and to further evaluate the reliability of the suggested tool. 

## Figures and Tables

**Table 1:** Mean values of rectum and breast physical and radiobiological equivalent doses for different *α*/*β* ratios.

	*D* (Gy)	EQD_2_ (Gy)
Rectum	63 (*α*/*β* = 10)	67 (*α*/*β* = 10)

Breast	55 (*α*/*β* = 2.3)	63 (*α*/*β* = 2.3)
	55 (*α*/*β* = 10)	58 (*α*/*β* = 10)

**Table 2:** Patients' characteristics and EORTC/RTOG acute toxicity for prostate (rectal toxicity) and breast (skin toxicity) cancer.

Prostate cancer (*N* = 50)					
Median age (range)	70 (63–78)				
T1 (%)	19/50 (38%)				
T2 (%)	31/50 (62%)				
Mean PSA (range)	8.142 (6.5–9.9)				

EORTC/RTOG rectal acute toxicity					
	Grade 0	Grade 1	Grade 2	Grade 3	Grade 4

		Increased frequency or change in quality of bowel habits not requiring medication/rectal discomfort not requiring analgesics	Diarrhea requiring parasympatholytic drugs/mucous discharge not necessitating sanitary pads/rectal or abdominal pain requiring analgesics	Diarrhea requiring parenteral support/severe mucous or blood discharge necessitating sanitary pads/abdominal distention	Acute or subacute obstruction, fistula, or perforation; GI bleeding requiring transfusion; abdominal pain or tenesmus requiring tube decompression or bowel diversion
*N* (%)	35/50 (70%)	14/50 (28%)	1/50 (2%)	— (0%)	— (0%)

Breast cancer (*N* = 50)					
Median age (range)	56 (44–72)				
T1 (%)	35/50 (70%)				
T2 (%)	15/50 (30%)				
Menopausal (%)	43/50 (86%)				

EORTC/RTOG acute skin toxicity					
	Grade 0	Grade 1	Grade 2	Grade 3	Grade 4

		Follicular, faint, or dull erythema/epilation/dry desquamation/decreased sweating	Tender or bright erythema, patchy moist desquamation/moderate edema	Confluent, moist desquamation other than skin folds, and pitting edema	Ulceration, hemorrhage, and necrosis
*N* (%)	13/50 (26%)	13/50 (26%)	19/50 (38%)	5/50 (10%)	— (0%)

**Table 3:** Spearman's rho nonparametric test for the correlation between *D*_50_ (Gy), *V*_60_ (%), and acute rectal toxicity according to RTOG/EORTC criteria.

Spearman *ρ*	*V*_60_ (*α*/*β* = 10 Gy)	RTOG
*D*_50_ (*α*/*β* = 10 Gy)	rho = 0.408 *P* = 0.003	rho = 0.514 *P* < 0.001
*V*_60_ (*α*/*β* = 10 Gy)		rho = 0.469 *P* = 0.001

**Table 4:** Spearman's rho nonparametric test for the correlation between *V*_≥60_ (%) and skin toxicity according to RTOG/EORTC criteria.

Spearman *ρ*	RTOG
*V*_≥60_ (10 Gy)	rho = 0.616 *P* < 0.001
*V*_≥60_ (2.3 Gy**)**	rho = 0.931 *P* < 0.001

**Table 5:** Percentage of patients with dosimetric parameters within the international dose constraints [32, 33].

		Patients (%)	
	*D*_50_ < 50 Gy	*V*_60_ < 35%	*V*_≥60_ < 40%
Prostate	50 (100%) *α*/*β* = 10 Gy	50 (100%) *α*/*β* = 10 Gy	—
Breast	—	—	47 (94%) *α*/*β* = 10 Gy
	—	—	38 (76%) *α*/*β* = 2.3 Gy

**Table 6:** Previous studies for radiotherapy induced acute toxicity in terms of breast irradiation [34].

Authors	Patients	Dose/fraction (Gy)	Acute toxicity ≥G2 (%)
Storey et al. [35]	189	70–78*; 2	—
Fiorino et al. [36]	245	70–78; 2	—
Greco et al. [37]	135	76; 2	—
Peeters et al. [38]	641	78; 2	—
Kupelian et al. [39]	770	70; 2.5	9
Kuban et al. [40]	301	70–78; 2	—
Vavassori et al. [41]	1123	≥70; 2	25.1

*Two arms: first arm treated with conventional “box technique,” dose, 70 Gy; second arm treated with 3D conformal, dose, 78 Gy.
